# Advancements in the use of AI in the diagnosis and management of inflammatory bowel disease

**DOI:** 10.3389/frobt.2024.1453194

**Published:** 2024-10-21

**Authors:** Dalia Braverman-Jaiven, Luigi Manfredi

**Affiliations:** Division of Imaging Science and Technology, School of Medicine, University of Dundee, Dundee, United Kingdom

**Keywords:** inflammatory bowel disease, optical colonoscopy, deep learning, artificial intelligence, databases

## Abstract

Inflammatory bowel disease (IBD) causes chronic inflammation of the colon and digestive tract, and it can be classified as Crohn’s disease (CD) and Ulcerative colitis (UC). IBD is more prevalent in Europe and North America, however, since the beginning of the 21st century it has been increasing in South America, Asia, and Africa, leading to its consideration as a worldwide problem. Optical colonoscopy is one of the crucial tests in diagnosing and assessing the progression and prognosis of IBD, as it allows a real-time optical visualization of the colonic wall and ileum and allows for the collection of tissue samples. The accuracy of colonoscopy procedures depends on the expertise and ability of the endoscopists. Therefore, algorithms based on Deep Learning (DL) and Convolutional Neural Networks (CNN) for colonoscopy images and videos are growing in popularity, especially for the detection and classification of colorectal polyps. The performance of this system is dependent on the quality and quantity of the data used for training. There are several datasets publicly available for endoscopy images and videos, but most of them are solely specialized in polyps. The use of DL algorithms to detect IBD is still in its inception, most studies are based on assessing the severity of UC. As artificial intelligence (AI) grows in popularity there is a growing interest in the use of these algorithms for diagnosing and classifying the IBDs and managing their progression. To tackle this, more annotated colonoscopy images and videos will be required for the training of new and more reliable AI algorithms. This article discusses the current challenges in the early detection of IBD, focusing on the available AI algorithms, and databases, and the challenges ahead to improve the detection rate.

## 1 Introduction

Inflammatory Bowel Disease (IBD) causes chronic inflammation of the colon and digestive tract. The IBDs include Crohn’s Disease (CD) and Ulcerative Colitis (UC). In CD the inflammation appears in any part of the digestive tract while UC is localized in the colon (Fakhoury et al., 2014). The highest prevalence of IBD is focused in North America and Europe, however, it has been increasing since the beginning of the 21st century in South America, Asia and Africa which in turn, has substantial implications on the understanding of the pathogenesis of the disease (Ng et al., 2017). The incidence rates are 1.85–10.5/100,000 person-years for CD and 1.9–17.2/100,000 person-years for UC in Europe and 6.3–23.8/100,000 person-years for CD and 8.8–23.1/100,000 person-years for UC in North America. The prevalence rates are 28.2–322/100,000 person-years for CD and 43.1–412/100,000 person-years for UC in Europe, and 96.3–318.5/100,000 person-years for CD and 139.8–286.3 for UC in North America (Ng et al., 2017; Kumar et al., 2023). In the UK specifically, the incidence is 36/100,000 person-years, which translates to 66 people being diagnosed with IBD every day. The prevalence in the UK equivalates to 0.8% of the population, which translates to 1 in every 123 people having a diagnosis of IBD with UC being more common than CD. The prevalence is higher in Scotland and Northern Ireland than in England and Wales (UK, 2024a). There is currently no specific test for diagnosing IBD, so the diagnosis consists of a combination of blood and stool tests, endoscopic and cross-sectional imaging, and histological tests. Investigations should focus on markers of disease activity, signs of malnutrition and malabsorption, blood on stool and signs of iron deficiency. Patients who show these markings then need to have a colonoscopy with biopsies from diseased and healthy segments to establish the diagnosis (Maaser et al., 2019). In the UK, more than half the patients who require a colonoscopy have to wait for more than 6 weeks, with one third waiting over 13 weeks for the procedure, leading to late diagnosis (UK, 2021). While there is no endoscopic feature to identify between the types of IBD, CD is characterized for showing discontinuous lesions while UC’s lesions are continuous (Maaser et al., 2019). There is no cure for IBD and no specific treatment, however, anti-inflammatory drugs such as 5-aminosalicylic, infliximab or thalidomide are commonly used as treatment combined with immunomodulators to regulate the immune system (Fakhoury et al., 2014). Every patient reacts differently to the treatment therefore the treatment should be personalized based on the symptoms and the severity of the disease (Seyedian et al., 2019). While the drugs help stop the progress of the disease, it is estimated that 50%–80% of patients with CD and 10%–30% of patients with UC require a colectomy (removal of the large intestine) over their lifetime (Khoudari et al., 2022). Diagnosing and treating the disease at its early stages leads to better clinical outcomes (UK, 2024b).

The applications of artificial intelligence (AI) in IBD include genomic data analysis, risk prediction models, diagnosis, treatment and management of the disease (Diaconu et al., 2023). Many reviews are being published on the use of AI for the diagnosis, monitoring, and treatment of IBD. However, databases are a very important aspect in developing this technology, and this review focuses on the public datasets currently available for the development of deep learning image recognition algorithms for colonoscopy images, particularly in diagnosing IBD, classifying UC and CD and monitoring the progress of the disease, and provides a review on the current research in the field and the challenges moving forward.

## 2 Medical procedure

The optical colonoscopy procedure is performed by an expert colonoscopist, the training needed to become competent in colonoscopy is extensive, requiring at least 175 procedures. Experienced colonoscopists have an important role in reducing pain and discomfort for the patient and identifying lesions (Manfredi, 2021). Before the procedure, the patient is required to follow a strict diet and drink a polyethylene glycol (PEG) solution to prepare the colon for screening. The adherence of the patient to the preparation guidelines is essential for the success of the procedure in IBD patients, as optimal bowel preparation improves the detection of non-polypoid flat lesions (Iacucci et al., 2019). The procedure starts with the patient lying sideways, the colonoscope is inserted through the anus to the rectum and sigmoid colon. The colonoscope is navigated through the colon to the cecum by applying a force and torque to the device. The mucosa is then inspected as the colonoscope is withdrawn, in this phase, the lesions are identified, and biopsies are collected as necessary. The 10 cm tip of the device provides two more degrees of freedom with a range of motion of 180° controlled using two wheels to navigate and examine the colon. The procedure is uncomfortable and painful for the patient, and while sedation can be administered, that would require a longer stay in the hospital for the patient and an increase in cost for the medical institution, hence the importance of performing a successful procedure identifying all the lesions present.

## 3 Optical colonoscopy in the detection of IBD

In the UK, the diagnosis of IBD can take from 6 months to 5 years after the appearance of the first symptom (UK, 2024a). A delay in diagnosis of CD is associated with an increased risk of stricturing, penetrating disease and intestinal surgery and in UC is associated with a higher risk of colectomy (UK, 2024b). Performing an optical colonoscopy (OC) is essential in patients showing signs and symptoms of IBD, as it provides real-time optical images of the bowel and allows for tissue samples to be obtained (Spiceland and Lodhia, 2018). It is not only used to diagnose and classify IBD but also to assess the response to therapy and survey for dysplasia, stricturing or malignancies, as patients with IBD have a higher risk of developing Colorectal Carcinoma (CRC) (Shergill et al., 2015). OC is the best tool in distinguishing between CD and UC before starting therapy, while there is no specific way to differentiate between the two, CD is consistent with skip lesions, rectal sparing and involvement of the terminal ileum while UC’s findings include diffuse and continuous inflammation proximal to the canal, granularity and superficial ulcerations. While OC is successful in identifying CD from UC in 89% of cases, 71% of patients need further tests such as histology, pathology or surgery to reach a diagnosis (Shergill et al., 2015; Terra Passos et al., 2018). There are a few scoring systems to aid in the diagnosis and classification of IBD the Mayo Endoscopic Subscore (MES), the ulcerative colitis endoscopic index of severity (UCEIS), the Crohn’s disease index of severity (CDEIS), and the simple endoscopic score for Crohn’s disease (SES-CD) (Daperno, 2023). As reported in Table 1: The Mayo Endoscopic Subscore is used to assess the severity of UC based on the appearance of lesions, with MES0 referring to inactive lesions and MES2 to moderate lesions. MES and UCEIS provide a score to assess the severity of UC based on the appearance of the most common lesions (vascular pattern, bleeding, erythema, erosions and ulcers) (Kim and Jang, 2013; Travis, 2024). The CDEIS and SES-CD require the physician to explore the five ileocolonic segments (rectum, right colon, transverse colon, left colon and ileum) and evaluate the lesions including ulcers, narrowing, inflammatory features and alterations in mucosa (Daperno, 2023). Despite the existence of these standardized scores, OC is a subjective procedure and is highly dependent on the expertise and sensitivity of the observer (Wang et al., 2020).

**TABLE 1:** Mayo endoscopic subscore.

Score	Disease activity	Endoscopic features
0	Normal or Inactive	None
1	Mild	Erythema, decreased vascular pattern, mild friability
2	Moderate	Marked erythema, absent vascular pattern, friability, erosions
3	Severe	Spontaneous bleeding, ulceration

As the incidence of colonic diseases increases so does the need for OC procedures, however, the amount of training a physician must complete to be able to perform a colonoscopy is extensive, including an average of 275 colonoscopies to become competent in the procedure (Sedlack et al., 2012). In patients with IBD, physicians performing the colonoscopy need to have proper training in the nature of the disease and specific colon preparation and understand the disease activity, as lesions can be flat, subtle and easy to miss, especially when stricturing or substantial disease activity is present (Iacucci et al., 2019). In the UK, a colonoscopy procedure costs the NHS about £650, additionally, patients with CD are associated with a mean cost of £6,156 per year including tests and treatment, this cost increases if the patient requires surgery (Lobo et al., 2020). An early and more accurate diagnosis leads to fewer tests and better clinical outcomes for the patients (UK, 2024a) which would lead to a decrease in costs for the NHS.

## 4 Current applications of AI in diagnosing and managing IBD

AI is revolutionizing healthcare, particularly the use of image recognition, as it is capable of analysing medical images aiding clinicians in the detection of subtle lesions, leading to a more accurate and early diagnosis of diseases (Ker et al., 2017). The performance of the model is dependent on the quantity and quality of the data used for its training. The data also must be properly annotated. Increased data annotation leads to more accurate and reliable models, however, as the amount of data increases data annotation becomes the biggest challenge in creating a reliable intelligent system (Cheng et al., 2018).

### 4.1 Current data availability

Since the accuracy in diagnosis using colonoscopy is highly dependent on the ability and expertise of the clinician, computer assisted diagnosis using AI is growing in popularity, especially in polyp detection. To achieve good performance of the AI models, high quality image and video sources are necessary (Brooks-Warburton et al., 2022). There are several image and video public datasets on colonoscopy studies, the most popular ones are Hyper-Kvasir and Kvasir-SEG. The Hyper-Kvasir dataset contains four main data records: labelled images (10,662 images), segmented images (1,000 images), unlabelled images (99,417 images) and labelled videos (374 videos which correspond to 889,372 video frames) and covers the entire gastrointestinal (GI) tract (upper and lower GI). It is used to detect 23 different lesions including polyps, caecum, and Barrett’s. It is the only public dataset that contains images related to UC, presenting 851 UC images from grades 0-3 (MES scale) but no images for CD (Borgli et al., 2020). Kvasir-SEG is based on the previous dataset, but it is solely focused on polyps. It contains 1,000 images in two folders: one for the annotated images and another for their masks (Jha et al., 2020). Even though, these datasets seem to have many images, most images are extracted from one video showing a simple polyp or lesion causing over 1,000 images to have no significant movement. Li et al. (2021) published another dataset from various sources to detect two categories of polyps: hyperplastic and adenomatous polyps. The dataset contains 155 videos (37,899 image frames) with their labels and bounding boxes. The dataset is divided at the video level in training, testing and validation sets (75%, 10% and 15%). Table 2 shows a summary of these findings.

**TABLE 2:** Publicly available datasets for OC images.

Dataset	Year	Field	Images
Hyper-Kvasir (Borgli et al., 2020)	2020	Lesion detection along the upper and lower GI, including UC.	10,662 labelled images, 1,000 segmented images, 99,417 unlabelled images, 374 unlabelled videos
Kvasir-SEG (Jha et al., 2020)	2020	Polyp detection	1,000 and masks
Li et al. (2021)	2021	Polyp classification: hyperplastic and adenomatous polyps	155 videos corresponding to 37,899 image frames

### 4.2 Current AI applications for polyp detection

**FIGURE 1:**
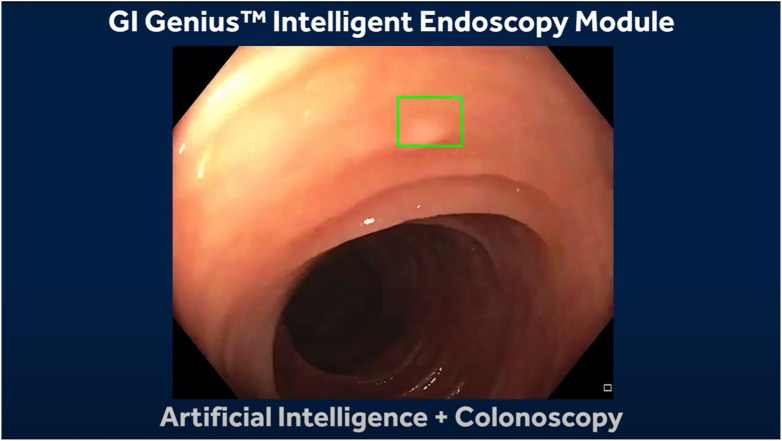
Medtronic GI Genius.

In OC, the development of AI models is mostly based on supervised data, where the annotations are created by expert endoscopists. These algorithms for colonic polyps aim to aid in detecting (CADe) miniature polyps that can be hard to miss and classification (CADx) between malignant and benign polyps by learning specific features such as mean vessel length, circumference, and brightness (Taghiakbari et al., 2021). The next challenge in polyp detection and classification with AI is their implementation into clinical practice, requiring regulatory approval and their acceptance by the medical staff and patients. These can only be tackled by demonstrating its accuracy and safety under real-time clinical conditions. Some healthcare and endoscopy manufacturers have developed algorithms for computer aided detection and computer aided diagnosis of colonic polyps and have been approved in Japan and Europe between the years of 2018 and 2020. Some examples are GI Genius by Medtronic (Figure 1), EndoBRAIN by Cybernet (Cybernet, 2018), ENDO-AID by Olympus (Olpympus, 2024), CAD EYE by (Fujifilm, 2024), DISCOVERY by Pentax (Medical, 2024) and Caddie by Odin Vision (Odinvision, 2024). Still limited research is available on the performance of these tools in OC (Mori et al., 2021).

### 4.3 Current AI applications for IBD

**FIGURE 2:**
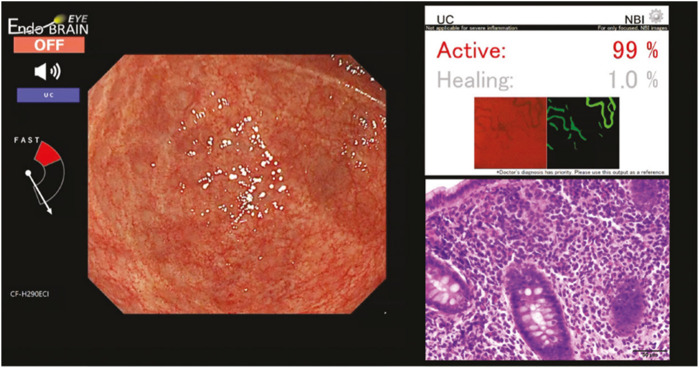
EndoBRAIN UC diagnosis for a MES1 lesion (Omori et al., 2024).

Despite the growing popularity and promising results of algorithms of image recognition in CRC, the application of deep learning algorithms for IBD is just starting. Yang et al. (2022) gives a review of nine studies using CNN for IBD detection. These studies were solely to assess on the prediction of prognosis and severity estimation of one type of IBD (eight studies for UC and one study for CD). Some studies used the public datasets already available while others gathered data from medical institutions. The studies performed with a sensitivity of 78% in UC and an accuracy of 65% in CD. With the growing popularity of the use of AI in colonic polyps, Cybernet released the EndoBRAIN-UC (Mori et al., 2021), a system that analyses features in the colonic mucosa such as capillary invisibility, dilation and hyperplasia using narrow band image to identify the presence of inflammatory lesions associated with UC was approved in Japan in 2020. Omori et al. (2024) conducted a study to compare the results of the AI system with the histopathological diagnosis of 52 patients and a total of 191 sites. 83.7% of the MES0 lesions were identified as “Healing” by the AI system and 92.9% of the MES2 cases were identified as “Active” lesions (Figure 2), concluding that the use of AI assisted diagnosis may reduce unnecessary biopsies in UC lesions, as no further samples would be required when the lesion is identified as inactive. This reduction in biopsies can decrease patient discomfort, reduce procedure time, and lower healthcare costs. However, the authors also discuss some limitations of their study. These include the limited number of patients, the system’s inability to contribute to the detection of dysplasia, and whether treatment interventions have an effect on the AI system’s accuracy. Additionally, the study highlights the need for further research to validate these findings in larger, more diverse populations and to assess the long-term impact of AI-assisted diagnosis on clinical outcomes. No similar system was found for the diagnosis and monitoring of CD. Sachan et al. (2024) reviewed 9 studies for the discrimination of CD and Tuberculosis, noting that both diseases exhibit similar symptoms as well as overlapping histological, radiological, and endoscopic findings. The studies use a CNN and Classification and Regression Tree (CART). Results show a sensitivity of 72.3%–100% and an accuracy of 69.59%–91.3%. Chierici et al. (2022) published a study on the use of a Residual Network to identify healthy, UC and CD colonoscopy images. A dataset of 14,226 images was used, classified as “positive” (diseased) and “negative” (healthy). The diseased images were then classified as UC, CD and IBD. Results were measured using the Matthews Correlation Coefficient (MCC), which is calculated using the true positive (TP), true negatives (TN), false positive (FP), and false negative (FN) values. The MCC ranges between −1 (complete misclassification) and 1 (perfect classification). The model achieved a MCC of 0.940 when classifying between IBD and healthy patients, 0.688 when classifying UC from CD and 0.931 when classifying UC and healthy individuals. There has also been some development in systems for evaluating dysplasia and other colonic lesions in patients with IBD, which is challenging due to the present inflammation in the colon. Vinsard et al. (2023) trained a previous CADe system with images from colonoscopic lesions (tubular adenomas, tubulovillous adenomas, adenocarcinomas, hyperplastic polyps and pseudopolyps) from patients with IBD. Results show a sensitivity of 67.4%, specificity of 88% and accuracy of 77.8% for chromoendoscopy images. Yamamoto et al. (2022) developed a system to identify high and low grade dysplasia in patients with IBD and evaluated it against experts, the model achieved a sensitivity of 72.5%, specificity of 82.9% and accuracy of 79%, higher than that of the experts. Table 3 shows a summary of the current studies using AI for IBD. Some reasons for the limited amount of research in using AI for IBD might be its low incidence compared to CRC, the difficulty in the diagnosis of IBD lesions solely by looking at the images and the limited amount of annotated data on IBD. These systems have attracted interest for their incorporation into clinical practice, however, the vast differences in datasets and methodologies used could limit their incorporation, and so far, there is a lack in data from patient outcomes (Chang et al., 2023). The ultimate goal of AI in IBD would be to decrease the time and amount of studies required for the diagnosis decreasing costs for health services and improving the patient’s quality of life (Sundaram et al., 2021; Chang et al., 2023).

**TABLE 3:** Comparison of AI studies on IBD.

Authors	Year	Field	Algorithm used	Measurable results
Yang et al. (2022)	2022	Prediction and prognosis of UC (8 papers) and CD (1 paper)	Deep learning convolutional neural networks	Accuracy: 78% in UC and 65% in CD.
Omori et al. (2024)	2024	Diagnosis and severity of UC	EndoBRAIN-UC	Accuracy: 83.7% in MES0 lesions and 92.9% in MES2 lesions
Sachan et al. (2024)	2024	Discrimination of CD and Tuberculosis (9 studies)	CNN and CART	sensitivity of 72.3%–100% and accuracy of 69.59%–91.3%
Chierici et al. (2022)	2022	Detecting CD and UC from colonoscopy images	Residual network	MCC 0.688
Vinsard et al. (2023)	2023	Detection of colonoscopic lesions in patients with IBD.	Scaling cross stage partial network (YOLOv4)	Sensitivity: 67.4%, specificity: 88% and accuracy: 77.8%
Yamamoto et al. (2022)	2022	Detection of high-grade and low-grade dysplasia	Deep convolutional neural network	Sensitivity: 72.5%, specificity: 82.9% and accuracy: 79%

## 5 Discussion

OC is used for the diagnosis of colon diseases including CRC and IBD as it provides real-time visualization of the colonic mucosa and allows tissue samples to be obtained for further analysis. However, it is highly dependent on the endoscopists ability and expertise, which leads to misdiagnosis, especially in small or hard to detect lesions (Iacucci et al., 2019). In IBD, a missed lesion can lead to a late diagnosis which is associated with an increased risk for stricturing, penetrating disease, surgery and even developing CRC (Uk 2024b). AI and DL algorithms have grown in popularity and are rapidly becoming more accurate and reliable, especially in the detection and classification of colonic polyps. The performance of these algorithms depends on the quality and quantity of the training data. For OC procedures, most publicly available datasets are focused on polyp detection and classification. There is limited public data available on IBD, with the HyperKvasir database being the only dataset found to have 851 images related to UC. This poses the biggest challenge in AI research related to IBD, as researchers have to spend a lot of time and resources gathering and annotating data to train the systems. For IBD, the research on using AI for its diagnosis is still in its infancy. Most of the research is based on UC, but there is a growing development in algorithms for identifying between UC and CD, assessing the severity of CD, and the detection of dysplasia, an early sign of CRC. An important need for this research to expand is a source of more annotated data on IBD, differentiating between CD and UC. One challenge in creating this data is the difficulty in distinguishing UC from CD solely by looking at the images and without considering the pattern and location of the lesions and histopathology results. With properly annotated data AI algorithms have the potential to reduce human error and automating the process of diagnosing IBD leading to an early diagnosis and better management of the disease, which in turn would improve the prognosis of patients with this disease.

The importance of AI in supporting the workforce is increasingly evident, as it potentially reduces the cognitive effort required to analyse images and potentially decreasing the time needed to perform the procedure. The integration of endoscopic robots (Manfredi, 2021; Manfredi, 2022) for screening purposes can further advance these algorithms, moving towards autonomous tasks in performing the procedure and analysing the colonic wall (Mathew et al., 2023a; Mathew et al., 2023b; Al-Bander et al., 2022). This advancement not only enhances efficiency but also ensures higher accuracy and consistency in diagnosing and managing colonic diseases. Furthermore, it can help reduce waiting lists and increase the early detection of diseases, ultimately improving patient outcomes.
